# Participatory Development and Analysis of a Fuzzy Cognitive Map of the Establishment of a Bio-Based Economy in the Humber Region

**DOI:** 10.1371/journal.pone.0078319

**Published:** 2013-11-07

**Authors:** Alexandra S. Penn, Christopher J. K. Knight, David J. B. Lloyd, Daniele Avitabile, Kasper Kok, Frank Schiller, Amy Woodward, Angela Druckman, Lauren Basson

**Affiliations:** 1 Evolution and Resilience of Industrial Ecosystems, Department of Sociology, University of Surrey, Guildford, United Kingdom; 2 Centre for Environmental Strategy, University of Surrey, Guildford, United Kingdom; 3 Department of Mathematics, University of Surrey, Guildford, United Kingdom; 4 School of Mathematical Sciences, University of Nottingham, Nottingham, United Kingdom; 5 Land Dynamics Group, Wageningen University, Wageningen, The Netherlands; Cinvestav-Merida, Mexico

## Abstract

Fuzzy Cognitive Mapping (FCM) is a widely used participatory modelling methodology in which stakeholders collaboratively develop a ‘cognitive map’ (a weighted, directed graph), representing the perceived causal structure of their system. This can be directly transformed by a workshop facilitator into simple mathematical models to be interrogated by participants by the end of the session. Such simple models provide thinking tools which can be used for discussion and exploration of complex issues, as well as sense checking the implications of suggested causal links. They increase stakeholder motivation and understanding of whole systems approaches, but cannot be separated from an intersubjective participatory context. Standard FCM methodologies make simplifying assumptions, which may strongly influence results, presenting particular challenges and opportunities. We report on a participatory process, involving local companies and organisations, focussing on the development of a bio-based economy in the Humber region. The initial cognitive map generated consisted of factors considered key for the development of the regional bio-based economy and their directional, weighted, causal interconnections. A verification and scenario generation procedure, to check the structure of the map and suggest modifications, was carried out with a second session. Participants agreed on updates to the original map and described two alternate potential causal structures. In a novel analysis all map structures were tested using two standard methodologies usually used independently: linear and sigmoidal FCMs, demonstrating some significantly different results alongside some broad similarities. We suggest a development of FCM methodology involving a sensitivity analysis with different mappings and discuss the use of this technique in the context of our case study. Using the results and analysis of our process, we discuss the limitations and benefits of the FCM methodology in this case and in general. We conclude by proposing an extended FCM methodology, including multiple functional mappings within one participant-constructed graph.

## Introduction

### Bio-Based Economy in the Humber Region

The Humber region surrounds the tidal estuary of the UK’s largest river system. It is a large active industrial area comprising a diverse set of industries ranging from the UK’s highest concentration of food processing industries to oil refining and chemical and bio-chemical production facilities. The port of Immingham is the UK’s largest by tonnage and, along with the other Humber ports of Grimsby, Goole and Hull, forms one of the largest and busiest port complexes in Europe. The estuary provides infrastructure for 20% of national gas landing and 27% of UK oil refining capacity [1]. The wider region is a net energy exporter and, due to the large number of coal-fired power stations and heavy industrial facilities such as steel making and cement production, the source of 27% of total UK 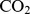 emissions emanating from industries subject to Integrated Pollution, Prevention and Control regulations [2] (This figure is based on a recalculation of 2008 Environment Agency IPPC data available from the reference and also available, on request, from the UK Environment Agency. Total commercial and industrial 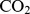 emissions in Yorkshire and the Humber, including IPPC and non-IPPC registered companies, was approximately 27 million tonnes (in 2007) (11% of total UK industrial 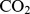 emissions). These figures are derived from DECC 2009 data also presented in the reference).

The estuary is of national and international biodiversity and conservation importance and due to climate change presents increasing flood risk management issues, both of which issues can cause friction over proposed development. Neighbouring communities face significant socio-economic problems including unemployment and fuel poverty. Development of the region is affected by, and affects, linked biophysical, industrial, economic, social and governance systems, populated by many diverse actors. The region faces significant new challenges and opportunities with transition to a low carbon economy and national energy security as current key and potentially controversial policy issues. It is one of the UK’s most important energy hubs, with strategic energy generation facilities and infrastructure, significant potential for carbon capture and storage and new investment in large-scale renewable energy technologies from offshore-wind to biofuels. The development of a bio-based economy has been recognised as a key opportunity for regional economic growth by regional industrial fora [1], [3]. This is due to both the presence of required infrastructure and support industries and also availability of feedstock from the substantial agricultural hinterland and bulk imports via the port. Numerous biodiesel and bioethanol facilities already exist or are under construction and the region expects to become the centre of an emerging UK biofuel industry responsible for 50% of UK production within the next five years. Significant investment is also underway in energy from biomass and biowaste alongside developments in biorefinery for high value chemicals.

As this sector emerges, managing interactions between policy, society, technology and economics within the system will be central in addressing the balance between economic development, efficient use of resources, reduction in environmental impacts and job creation on a regional and national scale. Hence decision making about the region and its possible future scenarios will have impacts on sustainability goals locally, nationally and globally. In this context we are using the development of a bio-based economy in the region as a case study to address the factors characterizing current key and potentially controversial, policy issues. By understanding the inter-relations between these factors and their consequent development we aim to provide decision support tools for the region to facilitate effective management of this transition. Although data obtained from academic and public sources will be invaluable in developing such an understanding, in this rapidly changing and highly regionally-specific context the input of expert stakeholders is vital. One particularly effective way to solicit such input is via participatory modelling; a process in which stakeholders collaborate in model framing and production.

### Participatory Modelling

Participatory modelling refers to any number of techniques by which stakeholders in a system of study are actively involved in some aspect of the creation or evaluation of models of that system. It is widely accepted that stakeholders can bring valuable first-hand knowledge (lay perceptions, expertise *etc.*) to a research process [4]–[7]. They can have meaningful ideas for selecting and developing a model, can help in collecting and integrating data, and can be involved in the development of scenarios, interpretation of results, and formulation of collective strategies or policy alternatives. On the other hand, engaging stakeholders is time-consuming, may bring plural perceptions to the research process rather than unambiguous data, may be difficult to manage and might be perceived to be difficult to carry out in research teams that are not interdisciplinary. Despite these potential pitfalls ‘participatory modelling, with its various types and clones, has emerged as a powerful tool that can (a) enhance the stakeholders’ knowledge and understanding of a system and its dynamics under various conditions, as in collaborative learning, and (b) identify and clarify the impacts of solutions to a given problem, usually related to supporting decision making, policy, regulation or management’ [8].

In many social domains, including our case study, data needed to construct a model may commonly be sparse, commercially sensitive or not centrally collected. In such situations engagement with stakeholders can increase the value of a research project by improving access to data and hence the reliability of the simulation emerging from it. Moreover, it may also improve the chances for implementation of a model’s results as stakeholders become more personally connected to and interested in the goals of the research. Our aims in running a participatory modelling exercise were thus twofold: to gather information about what a variety of local stakeholders considered to be key in understanding how a particular local industrial system will develop; and to enhance their understanding of, and engagement with, modelling and complexity approaches to their region.

Most participatory modelling techniques require extensive and ongoing engagement with stakeholder groups in order to iteratively frame, produce and refine a model of the system in question [9]. For example story and simulation [10], or companion modelling and participatory multi agent modelling approaches [5], [11]–[13]. For the most part stakeholders participate in framing and repeatedly evaluating detailed models of particular types produced by expert modellers, rather than being involved in producing models themselves. Other approaches, such as Bayesian belief networks for example [14]–[17], allow stakeholders themselves to be fully involved in model construction, but still require an extensive participatory process and good data availability or in depth empirical knowledge for determining conditional probabilities on system variables.

Due to the complex nature of our case study coupled with the scarcity of system data, we required a methodology which could capture qualitative knowledge from a variety of domains, social, economic, political, environmental and engineering. Additionally, given the very limited time that our stakeholders had available and our goals of increasing stakeholder engagement in and awareness of ‘whole systems’ or complexity approaches we chose to use a methodology in which the stakeholders themselves would be able to construct the model and which could produce preliminary results within the course of a one day workshop. After consideration of all these factors, the specific participatory modelling methodology which we chose to use for our initial approach to the case study was fuzzy cognitive mapping, or FCM.

### Fuzzy Cognitive Mapping

Fuzzy cognitive mapping was originally developed by Kosko [18] as an extension of Axelrod’s cognitive maps, which were designed to represent social scientific knowledge [19]. FCM has since been widely used for problem solving in situations in which numerous interdependencies are thought to exist between the important components or variables of a system, but quantitative, empirically-tested information about the forms of these interdependencies is unavailable [20]–[26]. The method aims to encapsulate the qualitative knowledge of expert participants or system stakeholders in order to rapidly construct a simple systems dynamics model of a specified issue. In the context of environmental management, it has been suggested that the technique is particularly useful in four types of situation [27]: firstly, when behaviour and decisions of stakeholders play an important role in determining the outcome of a system’s development; secondly when detailed local knowledge, but not scientific data, is available; thirdly in ‘wicked’ environmental problems, which are complex and have no ‘right’ answers; and finally, in problems in which public or stakeholder participation is desirable or required. All of these situations could be said to be true of our problem domain. The model produced via an FCM process is said to be *semi-quantitative* because the values of factors and the links between them can only be interpreted in relative terms [28]. Such a model can be used for projection or scenario testing purposes and to facilitate further discussion and interaction within or with a stakeholder group.

The process of model construction consists of several stages. Firstly the generation and selection by stakeholders of key *concepts* or *factors* which are important influences on, or parts of, the system of interest. Importantly, factors can be from any domain (social, economic, physical *etc.*) and may be qualitative or quantifiable. Secondly, discussion of, and decisions on, what the causal influences, or *links*, between factors are and whether they are positive or negative (that is, does an increase in one factor cause an increase or a decrease in a second factor to which it is causally connected). This allows the construction of a directed graph. Finally participants rank and verbally describe the strengths of these influences between factors, ultimately producing a directed graph with weighted links which we refer to as the *cognitive map* or FCM. This graph is then used as the basis for a simple model which is iterated forward to infer the possible, logical outcome of the system interconnections that participants have described, as well as the outcomes if links or their strengths are modified to represent alternative scenarios. FCMs may be generated collaboratively by a group of stakeholders at a workshop [28], [29], or elicited from individuals via questionnaires or interviews [27], [30]. Disparate maps of the same system from different sources can be combined and normalized [30]–[33]. Alternatively, conflicting structures resulting from different expert opinions, or different suggested policy interventions in the system, can be investigated as alternative scenarios [29], [34].

It is clear that any graph that stakeholders produce, collaboratively or singly, will be a representation of their own opinions and expertise about their system. The cognitive maps produced must therefore be explicitly understood as representing stakeholders’ subjective opinions on the area in question, with consequent potential differences between stakeholders from different domains. Maps may not represent reality, for example stakeholders may be sensitized to current controversial factors or infrequent, but high impact, factors which have recently occurred, and hence overestimate the number and weight of their connections [34]. The nature of this technique then, produces a potential weakness for quantitative modelling if the goal is to obtain a ‘definitive’ model via stakeholder interaction. It has significant strengths however, in its ability to engage stakeholders, promote learning and discussion amongst disparate groups, enhance understanding of whole systems approaches and extract a starting point for systems modelling where data on system structure is not available and where important variables are qualitative or hard to quantify [27], [30], [34]. Additionally, structural biases in the map or disagreements between experts give important information on stakeholders’ opinions, which can give a guide to points of intervention important for more socially effective policy or decision making in areas in which stakeholder involvement is crucial [29], [34]. Discussions of the causal ‘stories’ associated with the maps may also provide more subtle information on perceptions of how the system operates which can aid with future model development and engagement [34]. It must also be emphasized that what stakeholders in a system *believe* about its causal structure, and the effects of that structure, is in fact crucial to the decisions that they make, and hence to the *actual* structure and function of that system. This is true for any social system, but is particularly important in cases such as these when a stakeholder group includes key decision makers or when stakeholder participation is vital for successful decision or policy implementation. Despite their intersubjective nature therefore, FCMs and other participatory models have the potential to provide thinking tools that may change stakeholder behaviour and have a powerful impact on the system.

FCMs can be understood or used in different ways either as models of a system which can be used for decision support or for promoting organisational learning and discussion amongst stakeholders. In the context of much participatory work, the FCM is primarily an organisational learning tool and an aid to engagement. It is as valuable (or more) in its role in making explicit, then clarifying, mental models and provoking discussion amongst stakeholders as it is at providing a ‘definitive’ model of a given human system. The rapid construction of a simple mathematical model from such a cognitive map still serves an important function however, in making explicit to stakeholders what the consequences of their beliefs about lower level causal structure actually entail for the whole system. The benefit of using a mathematical analysis is to check the *internal consistency* of stakeholders’ cognitive maps of the system. If these maps are incorrect or incomplete, then an exploration using simple mathematical techniques can quickly expose potential inconsistencies with respect to the stakeholders’ own system knowledge and allow discussion, learning and clarification and redrawing of the map to more effectively represent their thinking. These models thus constitute an important part of the verification process. Standard methodology for producing a mathematical model from a fuzzy cognitive map is described below.

### Mathematical Model of the FCM

From the cognitive map produced at a participatory workshop, one would like to investigate the interaction of all the key concepts and links on a system in a systematic manner. Kosko [31] suggested using models drawn from neural networks as a means to mathematically explore the interactions of the concepts produced by experts. These models successively update each value of a concept using the previous value of the concept plus a sum of all incoming concept values and application of a thresholding function i.e., a step function centred at a half. The output of the model is a steady state from which a ranking of the most important factors in the system can be derived. Various different scenarios can then be tested and investigated on the system quickly and simply allowing participants of a workshop to develop a systems-level understanding of the implications of their mental models of the system in question. Subsequent research (see Hobbs *et al.* [23] and Mendoza *et al.* [25] for a review of FCMs) has focused on choosing different thresholding functions (ramp, sigmoid, *etc.*), introducing a weighted sum of the incoming concepts, and on learning algorithms for improving the weights used.

In this study we follow [23], [25], [26], [35] and turn the FCM produced by our workshop participants into a dynamical model
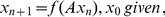
(1)where 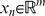, 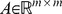 is the connectivity matrix created by participants, 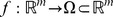 is the thresholding function, and 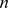 is the discrete time step. The state vector 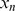 contains real values for all the key factors identified by participants. The weighted connectivity matrix 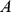 is formed by placing a value 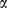 in 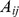 for every link from state 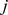 to state 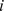. The value of 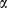 depends on the strength of the link and conventionally lies between −1 and 1. Although in principle there is no specific necessity for this restriction we choose to follow convention. In this paper we initially use a linear function; 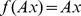 following the methodology described by [26], [28], [36].

The values of the states are usually interpreted in three ways; active/inactive, good/bad or important/not important [25], and when interpreting the results of (1), all three ways are used interchangeably given the ‘Fuzzy’ nature of the modelling. Hobbs *et al.* [23] suggest that for long-term policy decisions, the initial transient temporal dynamics are not of interest. This means that in most cases, one is interested in stable fixed points of (1) i.e., 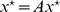 and 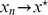 as 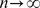. These stable fixed points allow one to rank the importance of the factors and establish dependences. This is useful information in evaluating and feeding back the model to the participants.

In the course of our participatory work in the Humber region, we ran both an FCM construction workshop and a follow-up verification exercise. For ease of explanation of the process on the day of the first workshop we made use of a linear mapping following [26], [28], [36]. For model production from the modified cognitive maps generated from our verification exercise we compared the results using both linear and sigmoidal FCMs, due to certain problematic properties of linear maps (See the Section on Comparison of Results of Linear and Sigmoidal FCMs). Using both of these functions gave us the opportunity to consider the sensitivity of our results to the functions used and to use this information to strengthen the verification process.

## Methods

### Humber Region FCM Workshop

During a one year period of engagement with regional industrial and political stakeholders, we identified potential study participants through a process of snowballing [37]. After in depth interviews with eighteen of these stakeholders, we decided to focus the workshop on the drivers and barriers surrounding the replacement of fossil fuel products by bio-based alternatives in the domains of energy, chemicals and food. The study involved data collection from primary sources (stakeholder interviews), but was considered to be exempt from the need for ethical clearance by the rules of the University of Surrey Ethics Committee for the following reasons: No deception was used in the research design; participants were not considered to be vulnerable; questions could not be deemed as sensitive or potentially offensive; there was no risk to volunteers’ health or wellbeing; no payments or benefits in kind were given to participants and issues of confidentiality and anonymity were guaranteed [38]. Participants were invited to interview with an email setting out the scope and aims of the exercise. Interviews were then carried out and recorded with the explicit, recorded, verbal consent of participants for the purposes of scoping the participatory research project and providing general information for model building. This is standard practice in this kind of participatory work, in which no ethical issues have been identified and which is carried out as a collaborative activity with stakeholders. An open, written, invitation was made to our contact lists and regional industrial fora and environmental managers groups. Eleven participants attended representing industry, local authorities and non-governmental organisations. During a day-long facilitated workshop participants produced a ‘cognitive map’ of interrelations between important factors in the development of a bio-based economy in the Humber region.

The workshop followed a standard form as follows:

1. Identifying Important Factors: Participants were first asked to make a list of factors (physical, political, social, and/or economic) that they considered important in the development of a bio-based economy in the Humber region.
2. Grouping the Factors: The factors identified were then grouped in relation to themes and system levels to consolidate the group’s ideas. The stakeholders then discussed the factors that arose, agreeing on 16 ‘dominant’ factors to focus on.
3. Linking the Factors: Participants discussed and decided on connections between the factors (relationships or edges) and directions of those connections (positive or negative influences).
4. Weighting the Links: Participants then ranked and defined the relative strengths of these interrelations according to a fixed scale (weak, medium or strong).
5. Creating the Model: The weighted graph produced was represented as an adjacency matrix which was used to update a vector of factor ‘values’, thus allowing a simple linear model to be rapidly produced and demonstrated on the day. Participants were then able to view and interrogate the model dynamically to evaluate different scenarios (see Section on Development of a Linear Model).

## Results

### Cognitive Map of the Humber Bio-based Energy System

The Map developed during the workshop consisted of 16 factors considered key for the development of the regional bio-based economy (See Table 1). The proportion of energy produced from bio-based as opposed to fossil sources was selected as a focal issue around which to construct the map and the directional, weighted, causal interconnections between factors were added starting from this point.

**Table 1:** Key factors generated by participants.

Index	Factors	Index	Factors
1	Infrastructure	9	Community Acceptance
2	Feedstock Availability	10	Technology Flexibility
3	Land Availability: Development	11	Ecological Sustainability
4	Supportive Legislation/Regulation	12	By-products
5	Finance & Funding	13	Existing Symbiotic industry
6	Competitiveness	14	National/International Instability (D)
7	Bio-Based Energy Production	15	Jobs
8	Knowledge	16	Fossil Fuel Price (D)

D indicates a driver.

**Figure 1:**
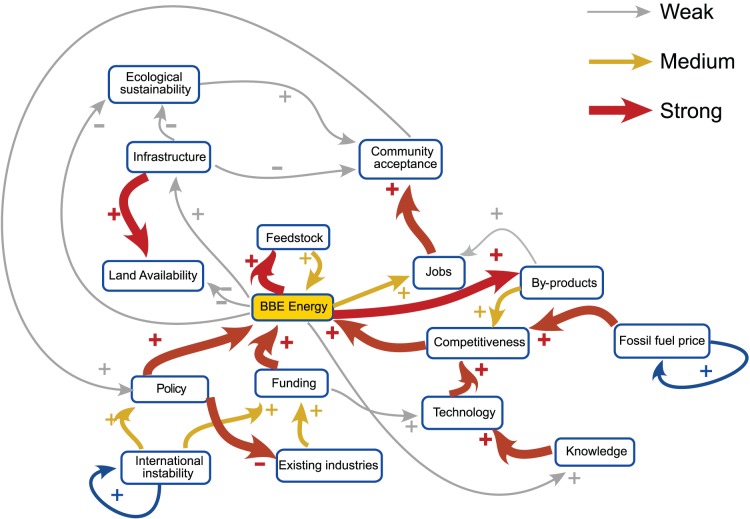
Humber region bio-based economy FCM from first workshop. Thickness of the links denotes the strength of the influence.

The cognitive map produced is illustrated in Figure 1. Several notable features are visible on a first examination. International instability (vs. UK stability) and fossil fuel price were identified as key external drivers of the regional system, a driver being defined as a factor with outgoing links only (These are denoted by self-reinforcing links). The map constructed consists of 3 relatively separate parts connected through bio-based energy production:

1. International instability and associated national funding and regulations that drives bio-based energy production
2. Competitiveness, oil price, and technology that drives bio-based energy production
3. Ecological sustainability, community acceptance, and infrastructure that reacts to changes in bio-based energy production but does not drive the system.

### Development of a Linear Model

Although the cognitive map is a useful starting point for discussion, we can interrogate the structure produced and the interaction of the key factors more effectively by constructing a dynamical model of the system. Following [23], [25], [26], [35] we turn the FCM into a dynamical linear model as described above in the Section on Mathematical Model of the FCM. The particular modifications used in our process are described below.

Within the weighted connectivity matrix 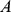 we have chosen 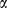 to be a slightly modified version of that found in [26]

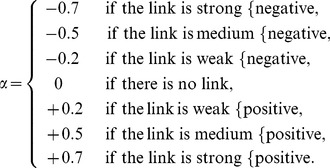
(2)


The modification that we make compensates for an ambiguity in the modelling process. Where a (positive) link is not simply strong or medium but is instead described as strong/medium we assign 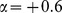; for instance the link between ‘Number of Jobs’ and ‘Community Acceptance’. Similarly a weak/medium link is given a weight of 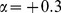 and a medium/weak link a strength of 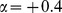.

One amendment also needs to be made to the weighted adjacency matrix before we use it in the linear FCM: 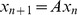. This change concerns the drivers of the system. As drivers (by definition) have no edges going into them, they will immediately be killed off (have value zero) under the first iteration of the linear FCM. To prevent this happening we provide the drivers with a self-reinforcing edge, a loop of strength one. So, for instance, concept 16 (fossil fuel prices) is a driver, so we set 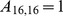.

Having made this change, all that is required to run the linear FCM is an initial condition, the iterative map can then be run to a fixed point. If we stipulate that the drivers initially have the same value, then the choice of initial condition does not effect the ordering of the concepts at the fixed point [39]. So we initially set the value of all drivers to one and the value of all other concepts to zero.

**Figure 2:**
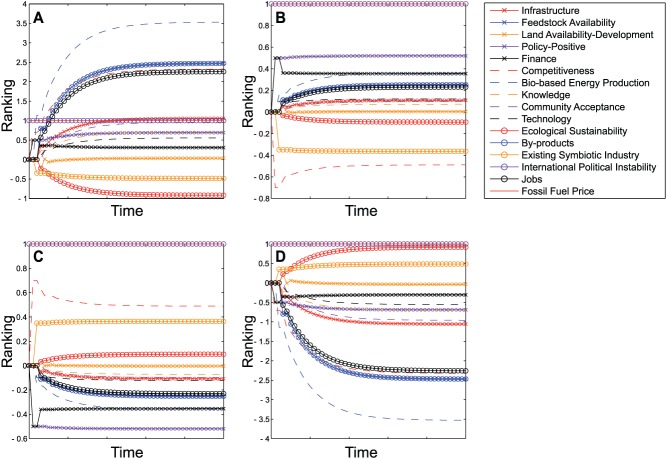
Output of the linear model of the FCM from the first workshop. a) Output from graph as drawn by participants, b)output showing the effects of reversing the effects of fossil fuel price alone, c) political instability alone and d) both fossil fuel price and political instability.

The time series output of the linear model produced from the original FCM graph was simulated and shown to the participants at the end of the workshop in order to provide a preliminary visual result. Participants were also shown output from models produced from the original graph with the effect of the two drivers reversed (that is, the signs of their outgoing links reversed within the adjacency matrix) both singly and simultaneously. All graphs were found to produce an output with a stable fixed point. Figure 2 shows the output of each of these cases. As discussed above, although the absolute values of factors are not meaningful, we can gain an understanding of the consequences of the factors’ inter-relations by considering their ranking at the fixed point.

In the original model as produced by the participants, bio-based energy production is seen to be maintained at a high level along with those factors on which it has positive causal influence, jobs, by-products and feedstock availability. Competitiveness is high with a consequent positive impact on bio-based energy production. This high production seems to be at the expense of sustainability, ranked lowest of the factors, existing symbiotic industries and land availability for development. When the effects of fossil fuel price were reversed bio-based energy production still maintained a relatively high, although diminished, ranking, as consequently do jobs, by-products and feedstock availability. Sustainability increases within the system whilst existing symbiotic industry remains low. The lowest ranked factor in this scenario becomes competitiveness, explaining the relatively decreased bio-based energy production. Reversing the effects of international instability produces a quite different result. Bio-based energy production, by-products, jobs and feedstock availability decrease much further in ranking as legislation and funding supporting bio-based energy production decrease. Sustainability increases in ranking whilst existing symbiotic industry and competitiveness become higher still. If the effects of both drivers are reversed then, in very marked contrast to our original scenario, bio-based energy production is driven down to become the lowest ranked factor. Excepting the drivers, which are maintained at 1, the factor rankings are reversed. By-products, jobs, competitiveness and feedstock availability are thus also low, with sustainability consequently becoming high.

According to this interpretation of the FCM graph, both drivers are required to maintain a high level of bio-based energy production, competitiveness and jobs simultaneously, although at the expense of sustainability. The influence of international instability and its associated group of factors is most crucial however as it contains two strong reinforcers of bio-energy production, favourable legislation and funding as opposed to the single, strong reinforcing factor of competitiveness associated with the fossil fuel-driven group.

In the context of the workshop discussion of these results, participants generally agreed that these would be expected outcomes and that the cognitive map effectively represented their thinking on the bio-based economy in a useful fashion. Due to time constraints on the day no further analysis or scenario exploration was possible at that time. For this reason a verification exercise was carried out as described below.

### Verification and Scenario Generation

An FCM is at its heart a representation of the opinions of a particular group of stakeholders on the causal structure of their system and as such cannot be separated from its intersubjective context. In many real situations it is impossible to define what the ‘right’ structure is and different stakeholders may hold different views on this. However, the limited time, specific participants and group dynamics of a workshop may bias the map produced in particular ways. In order to attempt to mitigate these biases and validate the map’s structure with a different group of experts, a feedback and verification exercise on the FCM was carried out at the local Environmental Managers Group. The group consists of environmental and technical managers from local heavy industries, as well as representatives of local authorities, network organisations and interest groups. There was a modest degree of overlap between participants in this second group and the original FCM workshop. The aim of this workshop was twofold. Firstly, to confirm that the structure of the map seemed reasonable to other local stakeholders with similar expertise and to determine whether any links had been overlooked in the first workshop. Secondly, to gather information on different potential scenarios for the region’s biobased economy which imply distinct, different causal structures and hence distinct differences in subsets of factors and links within the cognitive map.

Feedback, after a presentation of the methodology and results of the original workshop, was obtained from both an unstructured and a structured exercise. Firstly on a diagram of the original map, participants were asked to add additional links that they felt should be present and to delete or alter the weights of links which they felt should not be present or were incorrectly weighted. They were also invited to add additional factors or future factors and their links to the map and to comment on the rationale for changes that they had made. Secondly, they were given a structured questionnaire asking them to comment on the absence of particular links which we felt to be noteworthy based on our understanding of the system and its context. We present results from this first unstructured exercise.

**Figure 3:**
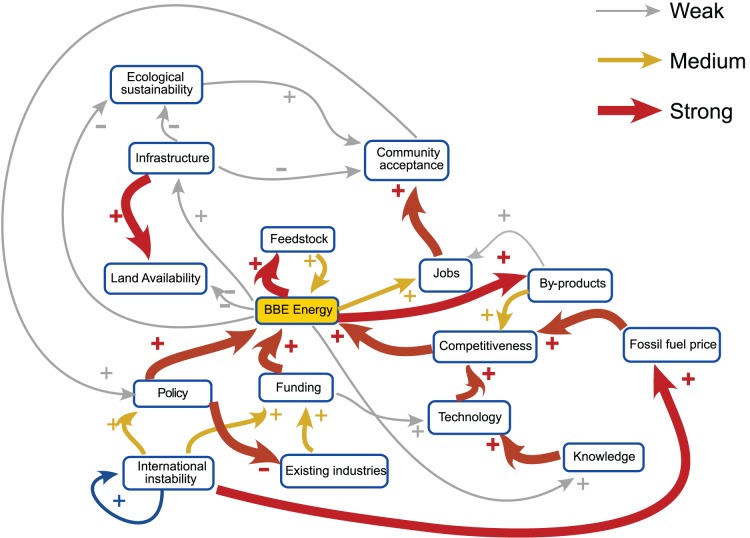
Modified FCM from the Humber Environmental Managers’ Meeting showing the addition of a link from international instability to fossil fuel price. Thickness of the links denotes the strength of the influence.

**Figure 4:**
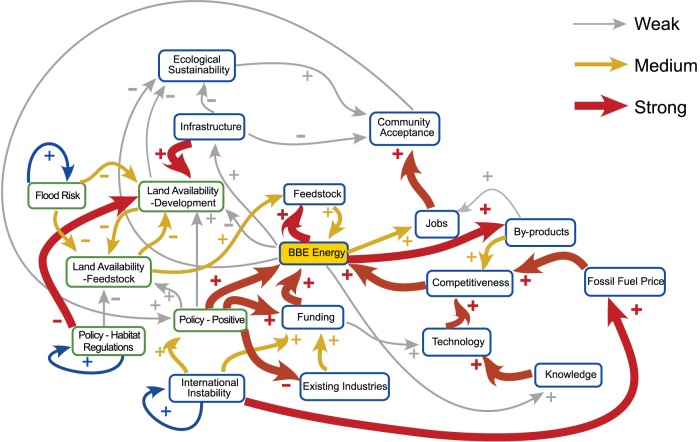
Modified FCM from the Humber Environmental Managers’ Meeting showing the locally grown feedstock scenario. Thickness of the links denotes the strength of the influence.

**Figure 5:**
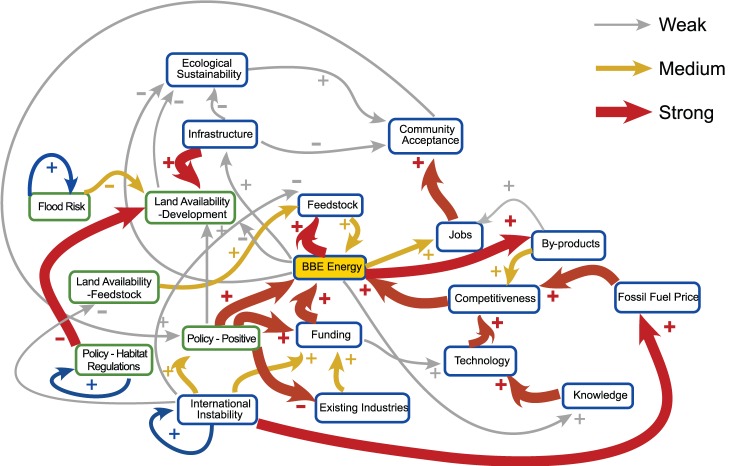
Modified FCM from the Humber Environmental Managers’ Meeting showing the non-local feedstock scenario. Thickness of the links denotes the strength of the influence.

In general the basic structure of the map was approved by participants with no suggestions to remove links, although different participants considered that a wide diversity of further causal connections should be represented in the map. The majority of responses agreed however that a connection should exist between international instability and fossil fuel price, meaning that fossil fuel price could no longer be considered a driver. We thus considered this to be a valid update to the map. Additionally, a large number of participants described and commented extensively on two potential scenarios for land use dynamics. Both included the addition of negative influences on the availability of land for development from policy (via habitat regulations) and from potential flood risk (a new factor). The first scenario also considered the possibility of locally-grown feedstock and consequent competition for land between industrial and agriculture use (Scenario 1), whilst the second scenario considered that feedstock would be imported from outside the system (Scenario 2). These scenarios were explicitly drawn out by stakeholders as possible alternative causal maps for the region under different possible futures and so are worthwhile to compare. The amended graphs illustrating each of these scenarios are illustrated in figures 3, 4 and 5.

### Analysis with Linear and Sigmoidal FCMs

In order to compare the results from our original workshop and aid with the verification process, we again construct dynamical models using the graphs produced by participants to form adjacency matrices. For this second round of model construction however, we decided to address problems that had become apparent in the use of a simple linear map. For our first workshop we followed the methodology and model production procedure described by Kok 2009 [28], which included the use of a linear mapping. This can easily be explained to non-expert participants and the update rule for each factor is just the weighted sum of all its inputs. It does however have certain problematic properties. In particular, it is possible for the value of the factors to become negative. The product of a negative factor and a negative link then needs to be carefully rationalised as it will evidently result in a net positive influence on the factor to which it is connected. Furthermore, the factor values may become large in magnitude and the weights taken in the connectivity matrix (2) may no longer distinguish between strong and weak links.

As mentioned briefly in the Section on Mathematical Model of the FCM there are several different functions that are commonly used in the construction of a mathematical model from the cognitive map (1). One such is a sigmoidal function, which may overcome some of the limitations of a linear mapping.

A sigmoidal mapping is given by using
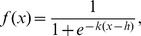
for constants 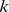 and 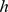 in (1). We take 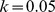 and 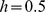 which guarantees that the map (1) has a unique (stable) fixed point (see [39] for a justification). Two slightly different procedures for implementing a sigmoidal FCM are described in the literature. The first is the same as the linear FCM, in that a 1 is put in the diagonal entry of the adjacency matrix for each driver. The second sets all the diagonal elements of the adjacency matrix to unity (see [35] for another example of the same procedure) and hence all the factors become drivers in the model. We chose to use this second methodology since we had not asked the participants in the workshop which factors they consider to be drivers (rather inferred them from their lack of incoming links) and all factors could potentially be maintained by influences external to the cognitive map.

The main advantage that the sigmoidal map possesses over the linear map is that the values of concepts are bounded (between 0 and 1). This means that the values of concepts cannot become negative and also that the effects of strong and weak links can always be distinguished. However the sigmoidal map also has some disadvantages; the main one being that the update rule is not as intuitive and harder to explain to participants (possibly leading to unexpected conclusions). Given the advantages and disadvantages of these two choices for 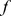, we compared the results of the original workshop, the updated map and the two scenarios under both a linear and sigmoidal mapping. This has the added benefit of allowing us to determine the sensitivity of our results to the form of the function 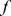, important given the previously stated aim of using these models to check the internal consistency of stakeholders’ cognitive maps.

For the updated map, with international instability and fossil fuel price linked, we used the original network as a basis and add a strong positive connection into the adjacency matrix from international instability to the factor representing fossil fuel price. For the linear FCM, this requires that we remove fossil fuel prices as a driver (removing its self-reinforcing link) as it now has incoming connections. Consequently we then use the initial condition of fossil fuel price zero, rather than the previous fossil fuel price of 1. Scenarios 1 and 2, locally and non-locally produced feedstock with habitat regulations and flood risk (which we will refer to as local and non-local), were combined one at a time with the updated network including a link from international instability to fossil fuel prices. In adding the scenarios we kept the base network the same and simply added the extra edges and concepts. In neither scenario did this cause international instability to cease to be a driver. However the additions created two new drivers in each scenario, Flood Risk and Policy - Habitat Regulations. As a result the initial conditions used were one for the concepts international instability, flood risk and policy - habitat regulations, and zero for all other concepts. Due to the addition of extra factors the maps for Scenarios 1 and 2 now contain 19 rather than 16 factors.

### Comparison of Results of Linear and Sigmoidal FCMs

**Figure 6:**
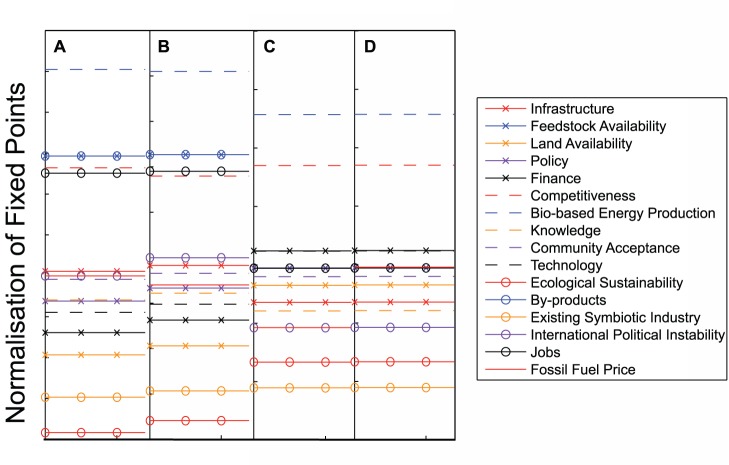
Results of adding in a link from international instability to fossil fuel price. Figures show ranking of factors at a stable fixed point using a linear map without (a) or with (b) the additional link, or using a sigmoidal map without (c) or with (d) the additional link.

When comparing the output of the linear and sigmoidal mappings applied to the same graph, it quickly becomes apparent that the functional form of the mapping may make a large difference to the results. Figure 6 shows the fixed points of the dynamical models (linear or sigmoidal) created from the original map from the first workshop compared with those produced from the updated map (Figure 3), in which international instability influences fossil fuel price. Using a linear mapping we can see that the ranking of the factors at the fixed point is changed only minimally, with principal factors of interest such as the level of bio-based energy production and ecological sustainability remaining unchanged in ranking, and jobs and competitiveness reversed in order with each other. Neither is significant change on adding the additional link apparent using a sigmoidal FCM. Adding the link from international instability to fossil fuel price leaves the ranking of the majority of factors, including bio-based energy production, competitiveness, ecological sustainability and jobs, unchanged. This suggests that the link between the two drivers has a minimal impact on the outcome for the system as a whole. However, the overall ranking of factors is changed by using a sigmoidal rather than a linear mapping. Although bio-based energy production remains highest ranked and ecological sustainability remains low (although not at the same rank) in both mappings, suggesting that these results do not depend on the form of 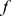, other factors undergo significant changes in rank. For example, finance rises from 13th in the linear mapping to 3rd under a sigmoidal mapping and land availability rises from 14th to 10th. These similarities and differences suggest the possibility of using comparison between the mappings as a form of sensitivity analysis. We shall expand on this below after presentation of our initial findings.

**Figure 7:**
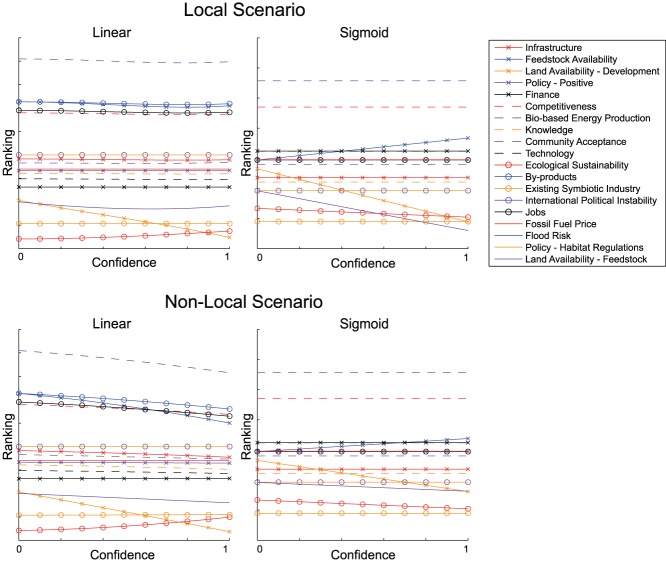
Results of phasing in the extra links and factors associated with the local and non-local land use scenarios. Figures show ranking of factors at a stable fixed point using either a linear or a sigmoidal map as a function of confidence (see text).

Figure 7 compares the local and non-local land use scenarios (as shown in Figures 4 and 5) analysed with both a linear and sigmoidal mapping. Rankings of factors are shown at the fixed point as before, but the specific sets of links and weights suggested by stakeholders for each scenario are gradually phased in and new fixed points calculated. ‘Confidence’ refers to the value of a multiplier on those new links from zero to one, thus the factor ranks at confidence zero are simply the ranks at the fixed point of the updated map. Ranks at confidence 1 are the relative values of factors at the fixed points of Scenarios 1 and 2, with weights as described by stakeholders.

Phasing in Scenario 1, local land use for feedstock, with a linear FCM changes little from the output of the base map. Land availability for development decreases in rank from 16th to 19th and ecological sustainability increases slightly, but other factors remain largely unchanged. Using a sigmoidal FCM, however, Scenario 1 gives rise to an increase in rank of feedstock availability, and significant decreases in land availability for both development and feedstock with consequent small increases in rank for ecological sustainability and existing symbiotic industry (defined in the workshop as existing industry supporting or connecting to bio-based industry). Land availability for feedstock maintains a lower rank than land availability for development as the confidence is increased.

Phasing in Scenario 2, non-local land use for feedstock, affects the ranking of more factors in the linear case than does Scenario 1. With a linear mapping we again see a decrease in land availability for development to the lowest ranking and a small increase in ecological sustainability. Presumably in both scenarios this change is caused by the new influence of habitat regulations. We also see a decrease in the ranking of feedstock availability and a slight decrease in ranking of jobs. It is notable that the absolute values at the fixed point of numerous factors are decreased by phasing in the new scenario, but without changing their ranking relative to others. Conversely, using a sigmoidal mapping, we see an *increase* in the availability of feedstock and again a decrease in land availability for development and feedstock. This is a similar result to the local scenario, however land availability for feedstock decreases at a lower rate, meaning that it is equally ranked with land availability for development when confidence is one.

It is noteworthy that Scenario 1 shows signs of the possible unexpected effects of a linear mapping discussed in the Section on Analysis with Linear and Sigmoidal FCMs. The change from decreasing to increasing value of some factors with increasing confidence can be traced back to the existence of a negative link between land availability for development and land availability for feedstock. As the value of land for development is driven negative by its strong negative link from habitat regulations, it will begin to have a *positive* influence on land availability for development and its connected factors. In Scenario 2, the two land availability factors, as well as existing symbiotic industry and sustainability, also have negative values at the fixed points. However in the map associated with this scenario, none of these negative factors have outgoing negative links. Such reversals in direction of influence as factors become negative, although potentially possible, certainly requires careful justification as it may radically change model output.

When we compare all four cases, two scenarios under two different functional mappings, it is clear that the functional form of the mapping may make an equal or even greater difference to the results than the scenarios themselves. For example feedstock availability increases under both scenarios with a sigmoidal mapping, but decreases or remains unchanged under a linear mapping. Consequently, if we consider possible interpretations of these results considering the mapping types one at a time we might draw quite different conclusions. With a linear mapping we might conclude that additional pressures on land availability from habitat regulations, flood risk and competition between land availability for development and feedstock make little difference to factors which concern us in the system as a whole (with the exception of land availability for development, which is significantly decreased by habitat regulations for only a small gain in ecological sustainability). Feedstock availability decreases only slightly in this scenario with no change in ranking for bio-based energy production, competitiveness or jobs. In the non-local scenario, land availability for development is again decreased by the impact of habitat regulations with a consequent increase in sustainability, but feedstock availability is significantly decreased. This is caused by the compound effects of new weak, negative links between the driver international instability and both land available for growing feedstock and feedstock availability, in a situation in which feedstock is mostly imported. Overall this does not lead to a decrease in rank for bio-based energy production however, although stronger links between feedstock availability and international instability might do so if they were present. The factor representing jobs decreases slightly to exchange its ranking with competitiveness, but both remain high. In the linear case then, we might conclude that bio-based energy production, jobs and competitiveness remain high whether feedstock is imported or locally-sourced. And that land availability for development is decreased whilst ecological sustainability is increased by the imposition of habitat regulations.

If we compare the two scenarios using the sigmoidal mapping we might conclude that again, key system indicators such as bio-based energy production, competitiveness and jobs are unaffected by whether feedstock is sourced locally or imported. We would conclude that, as might be expected, land availability for feedstock and development both decrease as the local land use scenario is phased in, as the land use types are now in direct competition with each other. With a sigmoidal mapping however we would conclude that both the scenarios of local and non-local feedstock production would lead to an overall *increase* in feedstock availability. This notable difference in results emphasises the value of comparing these two different functional forms. We can clearly see that the conclusion of whether feedstock availability increases or decreases is sensitive to the function 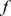, meaning that we need to look at these conclusions in more detail.

Such points of disagreement between the conclusions drawn from different functions may be used as a basis for further discussion and investigation. However, despite some large differences, there are certain similarities which are preserved in the results from individual maps treated with the two different functions. If we consider the application of the two functions as a form of sensitivity analysis we can have greater confidence in the results indicated by these similarities and hence draw preliminary conclusions. For example, in the analysis of the original map from the first workshop, there are five concepts which are in the top seven in both of the rankings (that from the linear FCM, and that from the sigmoidal FCM) and four which are consistently in the bottom seven. We say that these are the five most important concepts, and the four least important concepts respectively, to the development of a bio-based economy. The five factors in the top seven are bio-based energy production, by-products, feedstock availability, competitiveness and jobs. Similarly from our analysis, four of the least important of the stakeholders’ key concepts are ecological sustainability, existing symbiotic industries, land availability and knowledge. Community acceptance retains a moderate importance under both mappings. The same analysis performed on the updated map with the link between the two drivers would again suggest that bio-based energy production, feedstock availability, by products, competitiveness and jobs are the five most important factors and that knowledge, land availability, existing symbiotic industry and ecological sustainability are the least important factors. Community acceptance also remains relatively unchanged in position as a factor of moderate importance. Repeating this process for our two feedstock supply scenarios gives similar results: in the locally supplied feedstock scenario comparing the analysis of linear and sigmoidal FCMs suggests that bio-based energy production, competitiveness, by-products, feedstock availability and jobs are the five most important factors and that land availabilities for development and feedstock production, existing symbiotic industry, sustainability and knowledge are the five least important factors with community acceptance again relatively stable in a moderate position. In the non-local feedstock supply scenario, we have fewer certainties regarding important factors with only bio-based energy production, competitiveness and feedstock availability ranked as in the top eight under both mappings. However, the least important factors are more certain and remain unchanged as knowledge, ecological sustainability, existing symbiotic industry and land availabilities for feedstock production and development. None of the factors of particular importance to the bio-based economy seem particularly surprising. However, it is extremely interesting to note that stakeholders’ own models show the various forms of land availability to be relatively unimportant as this is a highly controversial issue in the region. Similarly promoting knowledge and training are thought to be particularly important to successful development of the area. Future participatory interrogation and possible re-interpretation of these results in the context of stakeholders’ own mental models of the system may either indicate a deficiency in the model or in stakeholders’ own perceptions of the system.

## Discussion

The models presented in this paper represent a first attempt in ongoing efforts to understand the bio-based economy in the Humber region and in this respect, despite their subjective nature, the cognitive maps produced by the group of expert stakeholders are in fact a highly useful output, both for us and the stakeholders (as evidenced by personal communication). The selection and verification of key factors, and the structure of their interactions, by a diversity of stakeholders provides a solid basis for further modelling work. Stakeholders identified a large number of factors supporting the development of a bio-based economy, with strong influences from policy, funding and fossil fuel price (via competitiveness). Connections which were perhaps more unexpected were also emphasised, for example from international instability to positive political and financial support for bio-based industries. Also perhaps unexpectedly, issues concerning ecological sustainability were considered to have only weak interactions with the rest of the system. Considerable variation of opinion amongst stakeholders, with regards to issues around feedstock availability and land use, was also revealed. Although in the initial workshop, discussion was eventually resolved with bio-based energy production driving feedstock availability without any negative influences, this was strongly questioned in the verification exercise and indeed in personal communication from stakeholders after the first workshop. The two different land use and feedstock source scenarios which were produced from the verification exercise demonstrate that a wide variety of opinion exists on the subject. Time limitations within the workshop meant that other key components of the bio-based economy, such as chemical and food production, were not considered. The potential for competition between these sectors for feedstock was thus not taken into consideration in model construction. It was however mentioned during our verification exercise as having an uncertain, but potentially important, impact on the evolution of the system.

The construction of the FCM and production of a dynamic output in the context of the initial participatory modelling workshop and subsequent verification exercise has additionally provided a means for our stakeholders to experience ‘systems modelling’ concepts and has increased project engagement. Feedback from participants has confirmed the usefulness of the process as a thinking tool for those involved. Despite this however, large discrepancies in results between the different mappings and different implementation procedures commonly used to create model output make interpretation of these simple models difficult. We compared the results from two different mappings, both of which have advantages and disadvantages (and neither of which can be considered as ‘correct’ due to the subjective and incomplete information used to create the model). The linear FCM is easy to explain to non-expert participants and rapid production of results and analysis can be carried out in a workshop context. However, the effect of factors becoming negative can profoundly change the model output and could be difficult to justify in many cases. It is likely that participants assumed that factors such as jobs or availability of land would be non-negative and would not have considered the implications of this possibility when constructing the interaction structure of the FCM graph. More qualitative factors such as, for example, community acceptance or ecological sustainability, or indeed factors such as price, could plausibly be modelled as either negative or positive. The effects of such factors could also justifiably be symmetric around zero. For example, if community acceptance of bio-based industry were to have a negative impact on habitat regulations, then we could justifiably expect community *dis*-acceptance (a negative factor value) to have a positive influence on the amount of habitat regulation. In the linear case, concepts are also unconstrained in magnitude. This could certainly be considered plausible when considering factors such as ecological sustainability or price, for which it might be difficult to assign particular upper or lower limits. Evidently the use of a linear mapping needs careful justification on a case by case basis.

The sigmoidal FCM does not have the same drawbacks as a linear mapping, as factor values are constrained to the unit interval and may correspond more closely to a functional response that participants might describe in some circumstances. However, since the sigmoidal FCM is nonlinear the analysis is significantly more difficult. It also requires additional parameters 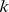 and 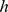 which arguably should be fixed by the participants. Only a limited amount of work has been done on comparing the use of different functional mappings in an FCM context. Tsadiras [40] discussed the appropriateness of binary, trivalent (in which factors can only take values of 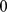 or 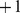 and 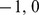 or 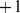 respectively) and sigmoidal FCMs for different situations. He concluded that binary and trivalent functions were useful in highly qualitative situations, whereas sigmoidal FCMs could be useful in both qualitative and quantitative problems and for strategic planning. No comparison was made between a sigmoidal FCM and any other continuous mapping. McNeil [41] discusses a wide range of ‘squashing functions’ (that is functions which constrain the factor values to between 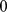 and 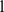) for use in FCMs and suggests different verbal labels which might be used to describe their effect. However he makes no mathematical comparison between either the functions or their use in model construction. A more extensive comparison of the implications of different functions on model output could certainly aid in the choice of function for participatory FCM construction and interpretation.

We have discussed the primary usefulness of FCM’s as representations of stakeholders’ beliefs and knowledge about a given system which have the additional strength of allowing a testing of the internal consistency of these beliefs. The production of a model and generation of output from a cognitive map makes explicit the consequences, both direct and indirect, of a given system structure and thus allows discussion, learning and re-evaluation of how this system may actually function. As such, it is vital that the conversion into a mathematical model represents the causal connections that stakeholders propose well enough to be able to perform these activities usefully. Yet, as we have shown, different commonly used FCM methodologies may have a larger impact on model output than changes in the structure of the cognitive maps themselves. As a first step towards overcoming this issue, a comparison of the output of the model generated using differing functional mappings could provide a form of sensitivity analysis as demonstrated above. Factors of interest which retain their approximate relative positions under different mappings could be considered as robust model output. Such an analysis is certainly useful in the ‘offline’ analysis of models outside a workshop context. It could also potentially be performed in a workshop setting with appropriate design of the feedback, although it might render understanding the output more difficult and hence discussion less productive.

When producing an FCM we must walk a fine line between keeping the map and model construction simple and understandable, yet producing output robust enough that participants in a workshop can usefully interrogate it and compare it to their own ideas about system function. The analysis of our case study and interaction with the stakeholders involved has suggested a possible methodological improvement which could meet both criteria. Given the differing nature of the factors within an FCM, it seems likely that for different factors and links within one network, different functional responses might more accurately represent the particular interactions. The process of constructing a linear FCM essentially forces participants to fit their system knowledge to a linearised version of reality. However, both during the course of the FCM workshop and via personal communications afterwards, participants suggested the possibility of non-linear mappings such as threshold functions for particular factors. This aspect of their expertise could represent a significant resource to be tapped in the construction of more useful models. Both this opportunity and the issues with standard functional mappings suggest a need to develop new methods which uncover and capture different functional relationships between factors beyond just strength and sign.

We propose an extension of standard FCM methodology, in which participants not only produce the factors and their interconnections, but choose from a set of possible functional mappings between factors for each link. A possible set of useful functions could include not only linear and sigmoidal functions, but tanh-like, step and Gaussian functions. Determining these functional responses in a participatory context would of course be challenging for participants and a significant part of developing this methodology would involve presenting the functions in intuitively understandable ways, as well as creating a tool kit to allow them to be added easily to the map (for example by the use of flashcards). Some of these functions would require additional information from the participants in order to specify them, for example the mid-point of a Gaussian, adding another level of detail. Additionally, to retain the facility for rapid generation of results, the production of dynamical output from the map during the course of the workshop would require the development of an easily-usable interface for the more complex modelling required. Participatory workshops such as these are often performed under time constraints and a two stage process of constructing a cognitive map and then addressing the issue of functional mappings might ensure better output and be more understandable to participants. Both mathematical and facilitative issues must be explored and resolved however, before such an extended methodology could be deployed.

## Conclusions

In summary, the fuzzy cognitive mapping exercise has produced major steps forward in our understanding of the potential development of a bio-based economy in the Humber region and in participants’ engagement with modelling and systems, and proved useful in promoting discussion of the issues involved. The work has given us large amounts of data on the significant factors and interrelations which we need to consider in constructing models of the Humber system. It has also revealed potential differing scenarios of land use and feedstock production which should be explored with further work. It has successfully provided what is perhaps the most useful aspect of the methodology, engagement and discussion within a group of disparate stakeholders and their co-construction of a systems-representation of their reality. These benefits should certainly not be underestimated and provide a solid platform for further work in the region, as well as benefits for the stakeholders themselves. Despite this, however, it has also revealed significant issues with the standard methodology used to create dynamic models of the FCM. Our analysis highlighted that different functional mappings commonly used to construct FCM output may give rise to large differences in the output and thus change the interpretation of different scenarios. Linear mappings in particular may give rise to results which affect the system output in ways that require careful justification and may be misleading. Sigmoidal mappings however, may not be appropriate for the interaction of all factors. This limits the usefulness of the approach when attempting to gain stakeholder feedback on model output in the context of a workshop. We suggest that a comparison between the output produced using different functions can act as a useful part of the validation process by highlighting which model outputs are more or less robust to the mapping used. In this case, bio-based energy production, competitiveness, by-products, feedstock availability and jobs were found to be the most important factors in the original and updated maps and in the locally-produced feedstock scenario under both mappings, whereas only bio-based energy production, competitiveness and feedstock availability were reliably important in the non-local feedstock scenario. Land availability (for feedstock production or development), knowledge, existing symbiotic industry and environmental sustainability were robustly found to be the least important factors in all maps, whereas community acceptance consistently retained a moderate ranking. Much work remains to be done to improve the methodology of using FCMs in a participatory context to produce more reliable mappings from stakeholders mental models to system-wide consequences of the interacting effects of the factors and interconnections that they describe. In pursuit of this goal, an expansion of the standard methodology has been suggested in which multiple functional relationships between different factors could be determined by participants and incorporated into a model. There will inevitably be numerous technical issues, both mathematical and in terms of participatory methods, which must be solved in order to develop this new approach. This work is under development in the course of our ongoing engagement with the Humber region and its stakeholder groups.
